# Supplementary material for: N-Glycosylation in Piroplasmids: Diversity within Simplicity
Source: Pathogens. 2021 Jan 8;10(1):50. doi: 10.3390/pathogens10010050 (PMC7826898; doi:10.3390/pathogens10010050)

Table S1. Identification of enzymes involved in the synthesis of the sugar nucleotides UDP-NAcGlc and GDP-Man in *P. falciparum* and the piroplasmids *B. bovis*, *T. equi* and *T. parva*. A scheme of these pathways is presented in Fig. S2. In these four parasites, UDP-NAcGlc is used as sugar donor for N-glycosylation. *T. equi*, additionally uses GDP-Man as mannose donor, while all use this sugar nucleotide in the GPI biosynthetic pathway. n.i.: not identified.

Abbreviation	Enzyme activity	*P. falciparum*	*B. bovis*	*T. equi*	*T. parva*
G6PI	Glucose-6-P isomerase	XP_001348515	XP_001609553	XP_004832357	XP_763365
GFPT	glutamine-fructose-6-phosphate aminotransferase	XP_001347529	XP_001609193	XP_004832549	XP_765503
GNA	Glucosamine-phosphate N-acetyltransferase	n.i.	n.i.	n.i.	n.i.
PAGM	Phosphoacetylglucosamine mutase	XP_001347982	XP_001611201	XP_004832899	XP_765493
UAP	UDP-N-acetylglucosamine pyrophosphorylase	XP_001350157	XP_001609008	XP_004829714	XP_766009
MPI	mannose-6-phosphate isomerase	XP_001349498	XP_001612088	XP_004831384	XP_764516
PMM	phosphomannomutase	XP_001347454	XP_001610907	XP_004833300	XP_766306
MPG	Mannose-1-phosphate guanyltransferase	XP_001348376	XP_001610191	XP_004829279	XP_765205
HK	Hexokinase	XP_966222	XP_001608748	XP_004830220	XP_765570

Table S2. Identification of three enzymes participating in the last steps of dolichol synthesis in piroplasmids. CPT: cis-prenyltransferase, homologous to *P. falciparum* PF3D7_0826400 (XP_001349261), containing a cis-IPPS Superfamily conserved domain (cd00475). PPRD: polyprenol reductase, homologous to *P. falciparum* PF3D7_1455900 (XP_002585471), containing a enoyl-CoA reductase conserved domain (cl28409). DK: dolichol kinase, homologous to *S. cerevisiae* SEC59 (NP_013726.1), containing a cytidylyltransferase domain (cl21502). CPT, PPRD and DK were annotated as hypothetical proteins or undecaprenyl diphosphate synthase, 3-oxo-5-alpha-steroid 4-dehydrogenase and dolichol kinase, respectively. (*) SEC59 homologues with no predicted conserved domain, likely due to sequencing mistakes.

Piroplasmid species	CPT	PPRD	DK
B. bovis	XP_001609396	XP_001611318	XP_001610276 (*)
B. bigemina	XP_012770149	XP_012768612	XP_012766127 (*)
B. ovata	XP_028868789	XP_028867790	XP_028867120
B. divergens	Bdiv_019050	Bdiv_019050	Bdiv_014590c
Babesia sp. Xinjang	XP_028871757	XP_028871510	XP_028871448
B. microti	XP_021337407	XP_012649685	XP_021338572
Theileria equi	XP_004832262	XP_004832719	XP_004829343
C. felis	CF004034	CF002172	CF000174
T. parva	XP_763441	XP_762780	XP_765268
T. annulata	XP_955106	XP_955357	XP_952012.1
T. orientalis	XP_009691469	XP_009692625	XP_009690727

Table S3. Predicted N-glycosylated proteins of *B. bovis*, T2Bo strain. Proteins predicted to be soluble, extracellular (**A**) or membrane-bound (**B**) and to bear one or more Asn-Xaa-Ser/Thr sequons, without Pro at Xaa, with high score (≥ 0.75 probability) are listed.

		**A**				
#**Accession**	#GI	*Babesia bovis* T2Bo	Positions of predicted N-glycosylation sites			
**XP_001608682.1**	156082395	hypothetical protein	80			
**XP_001608758.1**	156082547	DnaJ domain containing protein	46			
**XP_001609070.1**	156083172	spherical body protein 3	727	382	303	
**XP_001609122.1**	156083276	ribosomal protein L3 domain containing protein	81			
**XP_001609127.1**	156083286	hypothetical protein	130			
**XP_001609392.1**	156083817	hypothetical protein	608	551	374	328
**XP_001609486.1**	156084005	HAD superfamily hydrolase	236	82		
**XP_001609600.1**	156084234	hypothetical protein	391	62		
**XP_001609647.1**	156084328	hypothetical protein	790	259	14	
**XP_001609701.1**	156084436	hypothetical protein	57			
**XP_001609719.1**	156084472	hypothetical protein	129			
**XP_001609721.1**	156084476	mac/perforin domain containing membrane protein	28	24		
**XP_001609725.1**	156084484	hypothetical protein	345	195		
**XP_001609800.1**	156084634	membrane protein, putativve	40			
**XP_001610126.1**	156085298	subtilisin-like protein	601	289		
**XP_001610179.1**	156085539	hypothetical protein	362			
**XP_001610192.1**	156085565	ubiquitin-conjugating enzyme E2	63			
**XP_001610234.1**	156085649	hypothetical protein	183			
**XP_001610252.1**	156085685	erythrocyte membrane-associated antigen	154			
**XP_001610300.1**	156085781	SmORF	47			
**XP_001610337.1**	156085856	hypothetical protein	692	431	389	223
**XP_001610410.1**	156086002	hypothetical protein	452			
**XP_001610418.1**	156086018	hypothetical protein	276			
**XP_001610429.1**	156086040	cyclophilin	161			

**XP_001610441.1**	156086064	hypothetical protein	35	19				
**XP_001610577.1**	156086336	hypothetical protein	30					
**XP_001610672.1**	156086526	hypothetical protein	564					
**XP_001610684.1**	156086550	hypothetical protein	674	418	368	305	232	154
**XP_001610715.1**	156086612	SmORF	108					
**XP_001610737.1**	156086656	12D3 antigen	229					
**XP_001610783.1**	156086752	hypothetical protein	39					
**XP_001610828.1**	156086842	hypothetical protein	90	68				
**XP_001610832.1**	156086850	protein disulfide isomerase related protein	345					
**XP_001610846.1**	156086878	hypothetical protein	288	172	52			
**XP_001610959.1**	156087104	hypothetical protein	374	194	146	72	64	
**XP_001611015.1**	156087216	hypothetical protein	105					
**XP_001611063.1**	156087312	hypothetical protein	943	751	580	402		
**XP_001611104.1**	156087394	hypothetical protein	118					
**XP_001611109.1**	156087404	WD repeat domain containing protein	588	251				
**XP_001611301.1**	156087789	LytB protein	46					
**XP_001611368.1**	156087923	SmORF	59					
**XP_001611369.1**	156087925	SmORF	74					
**XP_001611539.1**	156088265	hypothetical protein	195					
**XP_001611693.1**	156088573	hypothetical protein	410	160	70			
**XP_001611710.1**	156088607	hypothetical protein	410	160	70			
**XP_001611725.1**	156088637	tRNA methyl transferase family protein	277	68				
**XP_001611782.1**	156088751	hypothetical protein	410	240	70			
**XP_001612129.1**	156089445	ubiquitin family protein	30					
**XP_001612214.1**	156089615	peptidyl-prolyl cis-trans isomerase, cyclophilin-type f domain containing protein	137	100				
**XP_001612255.1**	156089697	hypothetical protein	334	309				
			**B**					
#**Accession**	#GI	*Babesia bovis* T2Bo	Positions of predicted N-glycosylation sites					
**XP_001608872.1**	156082776	hypothetical protein	537					

**XP_001608971.1**	156082974	hypothetical protein	102						
**XP_001608980.1**	156082992	hypothetical protein	284						
**XP_001609412.1**	156083857	hypothetical protein	589						
**XP_001609788.1**	156084610	thrombospondin-related anonymous protein	273						
**XP_001609812.1**	156084658	p18 protein	785						
**XP_001610169.1**	156085519	hypothetical protein	930	327	252				
**XP_001610553.1**	156086288	GCC2 and GCC3 domain containing protein	853	816	497	473	234	229	142
**XP_001610606.1**	156086394	hypothetical protein	79						
**XP_001610777.1**	156086738	p-type ATPase	520						
**XP_001610968.1**	156087122	hypothetical protein	464	277					
**XP_001611503.1**	156088193	hypothetical protein	433	27					
**XP_001611871.1**	156088929	membrane protein	314	169					

Table S4. Transcription levels of N-glycosylation-related genes in *B. bovis* blood and tick stages. Data are expressed as log_{10} CPM. As reference, the CPM values for highly transcribed genes in merozoites (MSA-1) and kinetes (Kinete-specific, KS) are shown under "Reference genes"

Process	Gene id#	Genbank annotation of encoded protein	Common name	Log_{10} CPM	
				Blood	Tick
**N-glycosylation**	BBOV_IV000950	N-acetylglucosamine-1-phosphate transferase	Alg7	20.63	14.59
	BBOV_II005640	glycosyl transferase	Alg13	187.84	0.72
	BBOV_IV004090	conserved membrane protein	Alg14	3.38	4.02
	BBOV_II000220	oligosaccharyl transferase STT3 subunit	STT3	41.96	65.29
	BBOV_III003500	hypothetical protein	OST1	44.02	39.59
**sugar nucleotide synthesis**	BBOV_II000240	glucose-6-phosphate isomerase protein	G6PI	648.05	80.12
	BBOV_IV000250	glucosamine--fructose-6-phosphate aminotransferase	GFPT	493.68	112.73
	BBOV_III000660	phosphoglucomutase	PAGM	12.07	179.66
	BBOV_I003580	UDP-N-acetylglucosamine pyrophosphorylase	UAP	17.29	20.42
	BBOV_III009640	phosphomannose isomerase type I family protein	MPI	45.06	369.44
	BBOV_IV009850	phosphomannomutase	PMM	15.47	1086.53
	BBOV_II006730	mannose-1-phosphate guanyltransferase	MPG	23.83	51.13
	BBOV_I000860	hexokinase	HK	522.38	1166.98
**dolichol synthesis**	BBOV_IV002310	undecaprenyl pyrophosphate synthetase	CPT	17.97	22.34
	BBOV_III001830	3-oxo-5-alpha-steroid 4-dehydrogenase family protein	PPRD	44.02	39,.89
**reference genes**	BBOV_I003060	Merozoite surface antigen-1	MSA-1	5162.24	31.97
	BBOV_I002220	hypothetical protein	KS	12.66	243725.20

Figure S1. Predicted topology of *B. bovis* STT3. Red: inside; blue: outside; gray boxes; TM helix, IN-OUT; white boxes: TM helix, OUT-IN. “Inside” and “outside” predictions correspond to the cytoplasmic and luminal sides of the ER, respectively.

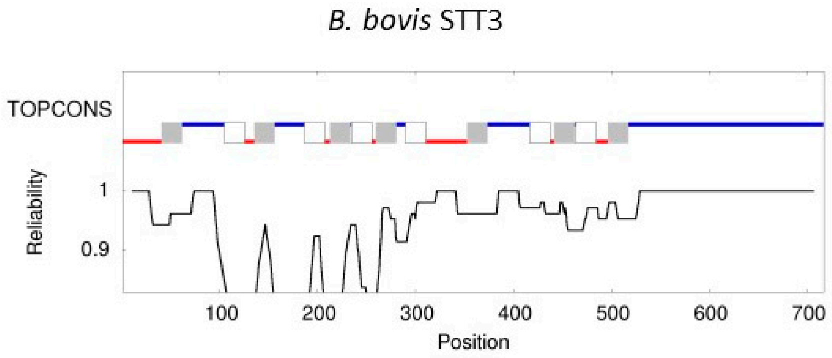

Figure S2. Predicted pathways for the biosynthesis of UDP-NAcGlc and GDP-Man in piroplasmids. *Babesia* s.s. use UDP-NAcGlc as sugar donor for N-glycosylation and GPI biosynthesis, and GDP-Man only for the latter process, while *T. equi* and *C. felis* use both sugar nucleotides in both processes. De novo pathways take place in the cytoplasm (in) starting from glucose-6-phosphate (Glu-6-P) and mannose-6-phosphate (Man-6-P), but salvage pathways for the incorporation of monosaccharides from the extracellular medium (out) are also predicted to take place. Precursors for the synthesis of UDP-NAcGlc and GDP-Man are shown in green and pink boxes, respectively. HK: hexokinase; G6PI: glucose-6-P isomerase; GFPT: glutamine-fructose-6-phosphate aminotransferase; GNA: glucosamine-phosphate N-acetyltransferase; PAGM: phosphoacetylglucosamine mutase; UAP: UDP-N-acetylglucosamine pyrophosphorylase; MPI: mannose-6-phosphate isomerase; PMM: phosphomannomutase; PMG: mannose-1-phosphate guanyltransferase (adapted from ref. 23). According to the presence of homologous genes in their genomes, piroplasmids use the same sugar nucleotide biosynthesis pathway.

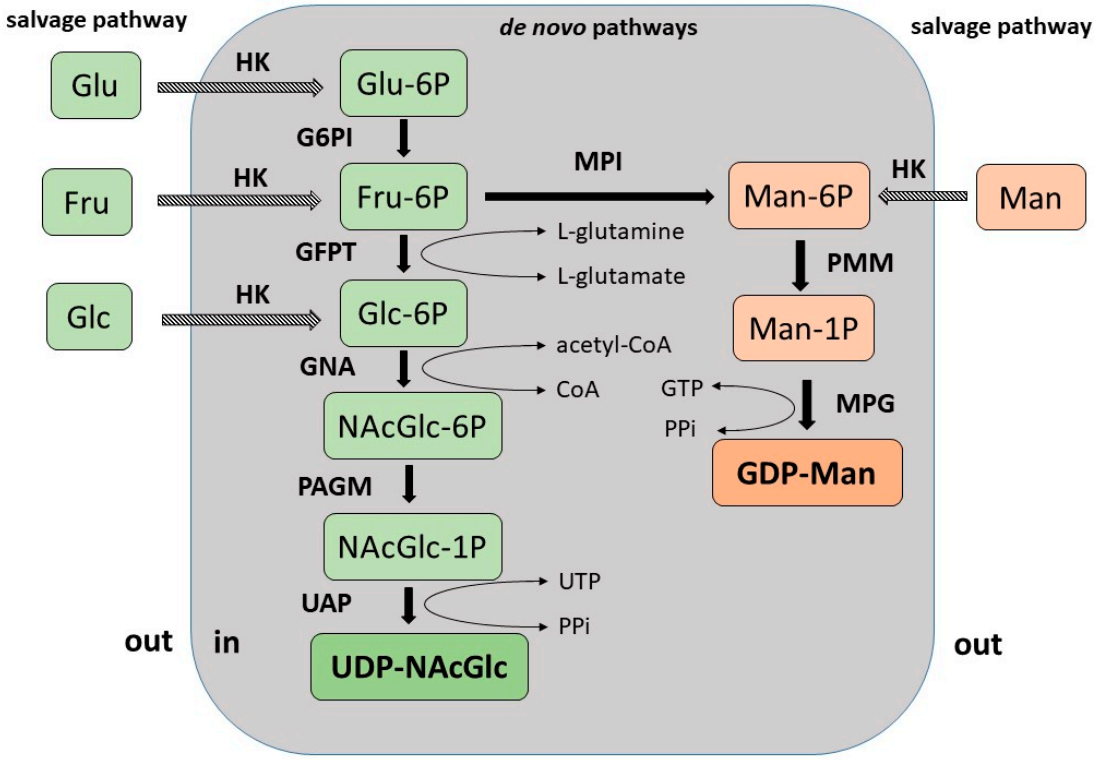

Figure S3. Number of predicted N-glycosylation sites in *B. bovis* secreted and membrane proteins. *B. bovis* proteins predicted to be exported or membrane-bound were analyzed for the presence of N-glycosylation sites (Asn-Xaa-Ser/Thr, without Pro at Xaa) with high score (potential >0.75). Only surface exposed sites were counted in the case of membrane proteins.

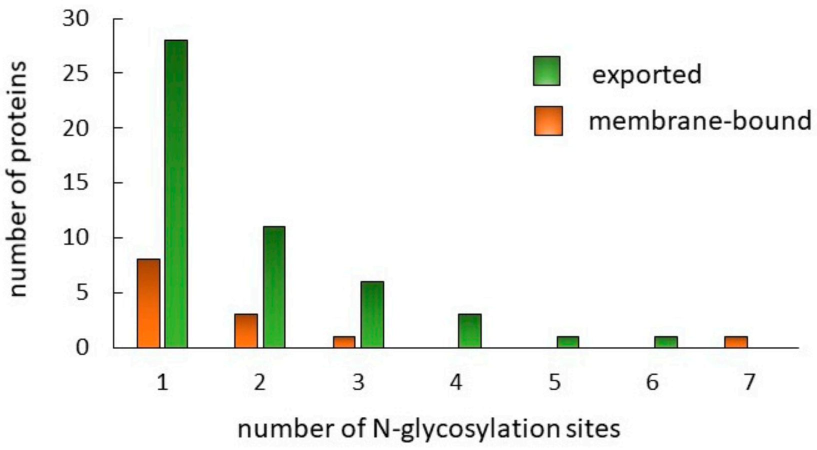

Supplement: Supplementary file 1 [file pathogens-10-00050-s001.pdf]
